# Study design of PANGAEA 2.0, a non-interventional study on RRMS patients to be switched to fingolimod

**DOI:** 10.1186/s12883-016-0648-6

**Published:** 2016-08-08

**Authors:** Tjalf Ziemssen, Raimar Kern, Christian Cornelissen

**Affiliations:** 1 Zentrum für klinische Neurowissenschaften, Klinik und Poliklinik für Neurologie, Universitätsklinikum Carl Gustav Carus Dresden, Technische Universität Dresden, Fetscherstr. 43, D-01307 Dresden, Germany; 2 Novartis Pharma GmbH, Roonstr. 25, D-90429 Nuernberg, Germany

**Keywords:** Multiple sclerosis, Relapsing remitting, Fingolimod, Efficacy, Safety, Modified Rio score, NEDA, No evident disease activity, Clinical routine

## Abstract

**Background:**

The therapeutic options for patients with Multiple Sclerosis (MS) have steadily increased due to the approval of new substances that now supplement traditional first-line agents, demanding a paradigm shift in the assessment of disease activity and treatment response in clinical routine. Here, we report the study design of PANGAEA 2.0 (Post-Authorization Non-interventional GermAn treatment benefit study of GilEnyA in MS patients), a non-interventional study in patients with relapsing-remitting MS (RRMS) identify patients with disease activity and monitor their disease course after treatment switch to fingolimod (Gilenya®), an oral medication approved for patients with highly active RRMS.

**Method/Design:**

In the first phase of the PANGAEA 2.0 study the disease activity status of patients receiving a disease-modifying therapy (DMT) is evaluated in order to identify patients at risk of disease progression. This evaluation is based on outcome parameters for both clinical disease activity and magnetic resonance imaging (MRI), and subclinical measures, describing disease activity from the physician’s and the patient’s perspective. In the second phase of the study, 1500 RRMS patients identified as being non-responders and switched to fingolimod (oral, 0.5 mg/daily) are followed-up for 3 years. Data on relapse activity, disability progression, MRI lesions, and brain volume loss will be assessed in accordance to ‘no evidence of disease activity-4’ (NEDA-4). The modified Rio score, currently validated for the evaluation of treatment response to interferons, will be used to evaluate the treatment response to fingolimod. The MS management software MSDS3D will guide physicians through the complex processes of diagnosis and treatment. A sub-study further analyzes the benefits of a standardized quantitative evaluation of routine MRI scans by a central reading facility. PANGAEA 2.0 is being conducted between June 2015 and December 2019 in 350 neurological practices and centers in Germany, including 100 centers participating in the sub-study.

**Discussion:**

PANGAEA 2.0 will not only evaluate the long-term benefit of a treatment change to fingolimod but also the applicability of new concepts of data acquisition, assessment of MS disease activity and evaluation of treatment response for the in clinical routine.

**Trial registration:**

BfArM6532; Trial Registration Date: 20/05/2015.

## Background

In recent years, the therapeutic options for patients with Multiple Sclerosis (MS) have steadily increased. Substances such as Fingolimod (Gilenya®) supplement the traditional first-line agents interferon (IFN) and glatiramer acetate, offering physicians the opportunity to optimize individual MS-treatment [1, 2]. The safety and tolerability profile of fingolimod and natalizumab is well understood. However, experience with new treatment options such as alemtuzumab, dimethylfumarate, and teriflunomide is limited in comparison to former approved substances, and especially data on the safety and tolerability of sequentially changed disease-modifying therapies (DMTs) are mostly not available. Since defined treatment algorithms for individual patients have not yet been developed, many MS patients may continue to receive suboptimal treatment for long periods of time.

To optimize treatment, a switch to a more effective medication generally needs to be considered if patients do not respond to or fail with their current therapy [2]. It is well accepted that the earlier in MS pathogenesis the therapy is adjusted (in the lower Expanded Disability Status Scale [EDSS [3]] range up to 3), the higher would be the benefit on long-term outcomes because MS progression might be more difficult to slow down at later stages [4, 5]. Since magnetic resonance imaging (MRI) parameters frequently assessed during therapy are sensitive markers to identify patients who are insufficiently responding to therapy [6], quantitative scoring systems incorporating relapses and MRI activity have been suggested as valuable diagnostic tools in clinical routine. Among them, Lublin et al. [7] defined disease activity at a particular time point on the basis of clinical relapses and MRI activity in the previous 12 months. Sormani et al. [8] modified the Rio score [9] to define treatment response based on relapse activity and MRI activity over a period of 1 year of treatment. However, the data underlying the modified Rio score was obtained from clinical studies on IFN-β [10], and the modified Rio score has not been evaluated for other therapies or under real-life conditions. Other scoring systems have been developed that assess parameters besides relapse and MRI activity, but there is currently no consensus among MS experts on the most sensitive measures applicable in clinical practice for identifying patients on suboptimal treatment [11, 12].

With the possibility to optimize treatment by sequentially applying novel and highly effective MS therapeutics, the MS community is increasingly accepting ‘no evidence of disease activity’ (NEDA) as an early objective for individual treatment. This new treatment paradigm is based on the view that the mere reduction of relapse rate and the attenuation of disease progression can no longer be accepted as sufficient in clinical routine. Therefore, NEDA was defined as no relapse activity, no EDSS progression, and no new MRI lesions (T1 Gd + and/or active T2 lesion; [13, 14]). Since these measures may not be able to address all aspects of the disease [7, 15, 16], brain volume loss (BVL) has been suggested as fourth NEDA measure (NEDA-4) to provide a more comprehensive and early picture of the focal and diffuse damage occurring in MS. MS experts recently proposed to further expand the current concept of NEDA to include neuropsychological aspects as well as other subclinical measures with a potential predictive value for treatment response [12]. In daily clinical routine, implementation of NEDA-4 as a treatment outcome goal, complemented by these subclinical measures might therefore offer the possibility of an early optimization of MStreatment [17].

Based on these considerations, we planned PANGAEA 2.0 (Post-Authorization Non-interventional GermAn treatment benefit study of GilEnyA in MS patients), a non-interventional study (NIS) to assess the benefits of a treatment change to fingolimod in patients identified as not responding to or having treatment failure with their current therapy. Fingolimod is approved in over 80 countries for the treatment of adult patients with rapidly progressing, severe RRMS or adult patients with high levels of disease activity despite treatment with at least one DMT [18–21]. As of Oct. 2015, it is estimated that fingolimod has been used to treat approximately 134,000 patients, summing up to a total exposure of over 265,000 patient years [22]. The well-established safety profile of fingolimod is currently being expanded by real-world data obtained during our predecessor study PANGAEA [23], which included RRMS patients who were either untreated or pre-treated with medications available at study initiation. Since further novel substances have subsequently been approved [24], PANGAEA 2.0 will provide additional and more comprehensive data on the safety of fingolimod in pretreated patients.

In this paper, we present the study protocol of PANGAEA 2.0 and propose a comprehensive, multidimensional approach for MS patient evaluation. By this approach, we will assess the long-term benefits of a treatment change to fingolimod in 1500 RRMS patients identified as being non-responders or failing their current first-line therapy. Disease activity at a given time point will be determined according to the criteria of Lublin [7] to identify sub-optimally treated patients. During a 3-year observational phase, treatment response to fingolimod will be evaluated by the modified Rio score [8] and by parameters that are based on both the treatment objectives of NEDA-4 [25] and the 2D Focussed Disability Scale (2D FDS) as part of our multidimensional approach for MS patient evaluation. The 2D FDS comprises clinical and subclinical measures representing both the patient’s and the physician’s perspectives. To handle the resulting amount of data and to assist neurologists in executing the complex processes required for MS diagnosis, treatment initiation, and long-term therapy, the software-based MS management system MSDS3D will be employed [23, 26]. Furthermore, a sub-study will assess the potential benefits of an independent analysis of routine MRI scans by a central reading facility. MRI analyses will additionally comprise quantitative MRI parameters such as brain lesion volume and brain lesion volume loss (BVL) that are generally not part of routine MRI data analysis. Consequently, PANGAEA 2.0 will expand the existing safety and efficacy profile of fingolimod and evaluate the clinical applicability of novel concepts for the evaluation of the patient status and treatment response in clinical routine [27]. PANGAEA 2.0 was started in June 2015 and is planned to continue until December 2019 in 350 neurological practices and centers in Germany, including 100 centers participating in the sub-study.

## Methods/design

### Study design

**Fig. 1:**
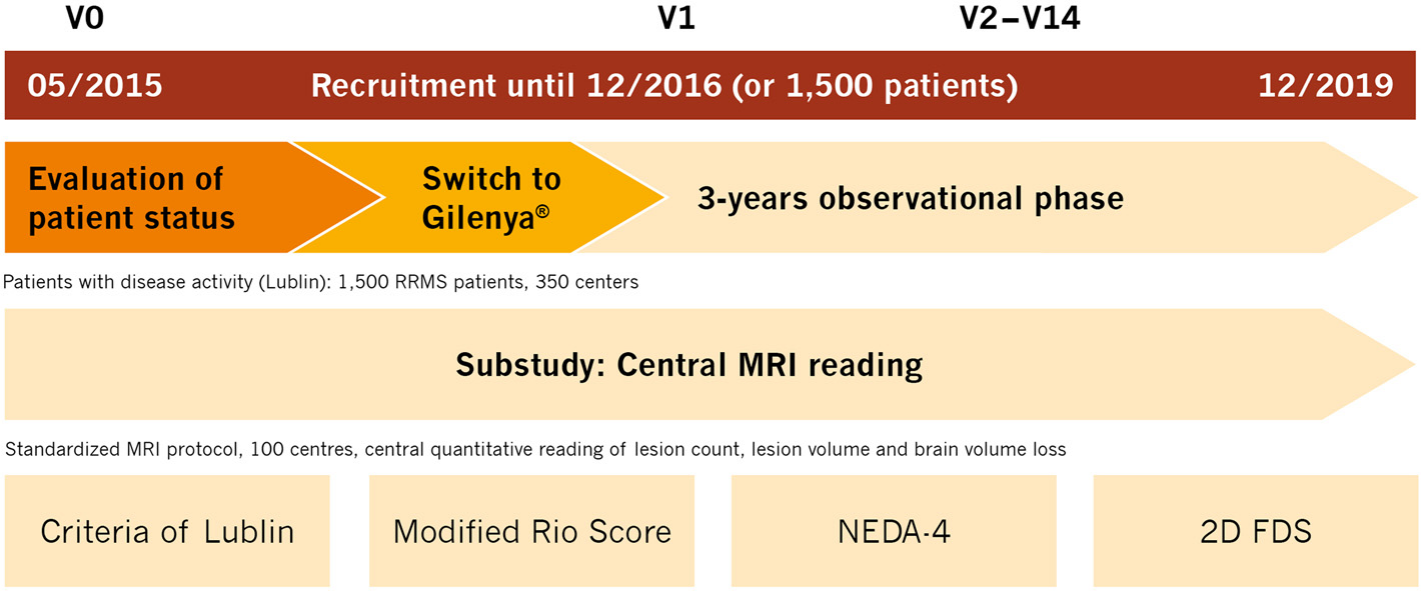
Study design of PANGAEA 2.0. The study involves an evaluation of disease activity to identify sub-optimally treated RRMS patients (V0), the switch of MS therapy to fingolimod (Gilenya®; V1), and a 3-year observational phase (V2–V14) to assess treatment response in several functional domains. A sub-study analyzes the benefits of a standardized quantitative evaluation of routine MRI scans by a central reading facility (NEDA-4: No Evidence of Disease Activity-4; 2D FDS: 2D Focused Disability Scale)

**Fig. 2:**
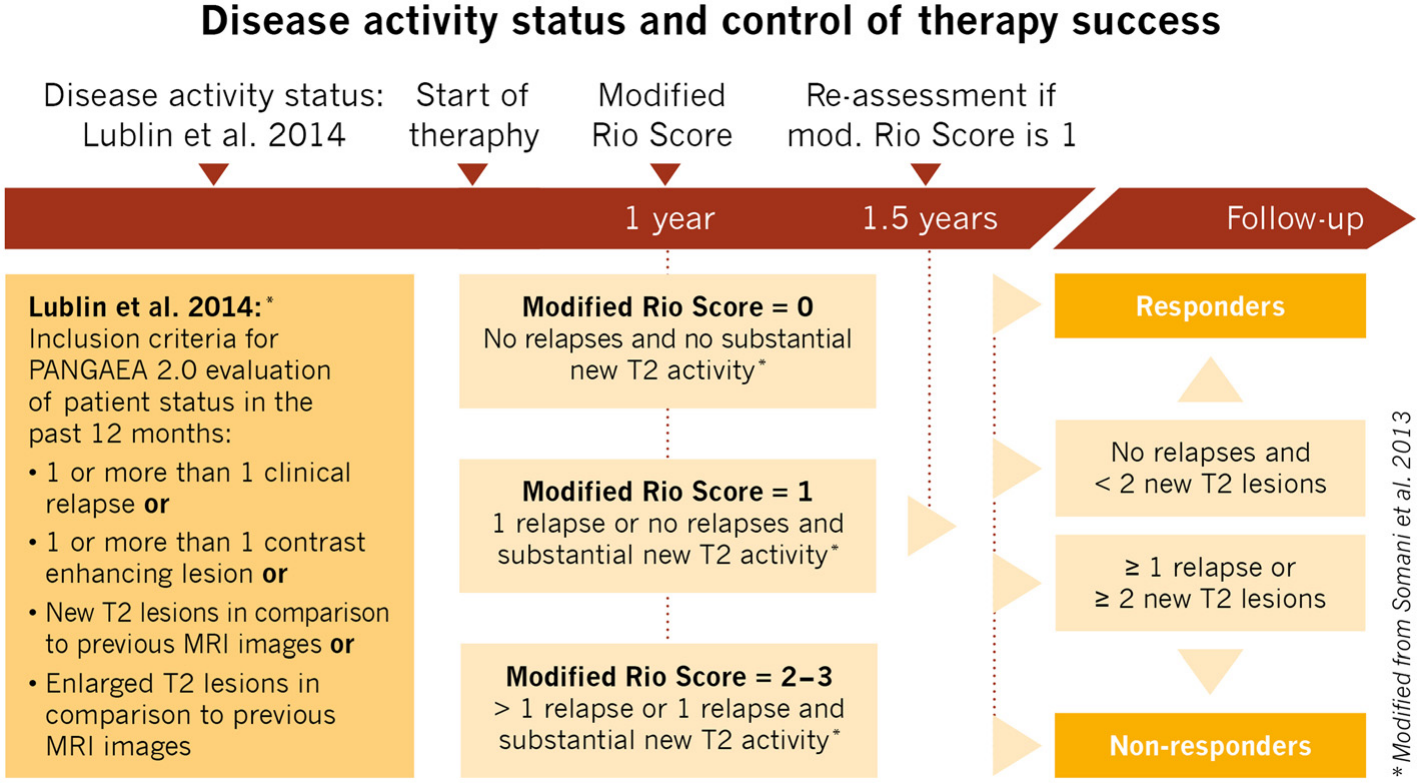
Assessment of disease activity and treatment response in PANGAEA 2.0. In the first study phase disease activity will be assessed according to Lublin et al. [7]. Disease active patients who switch to fingolimod are subjected to a 3-year observation period. Disease progression and treatment response will be assessed by using the modified Rio score [8] (figure adapted from [10]). Definition of active disease by Lublin et al. 2014 and evaluation of disease progression and treatment response by the modified Rio Score

**Fig. 3:**
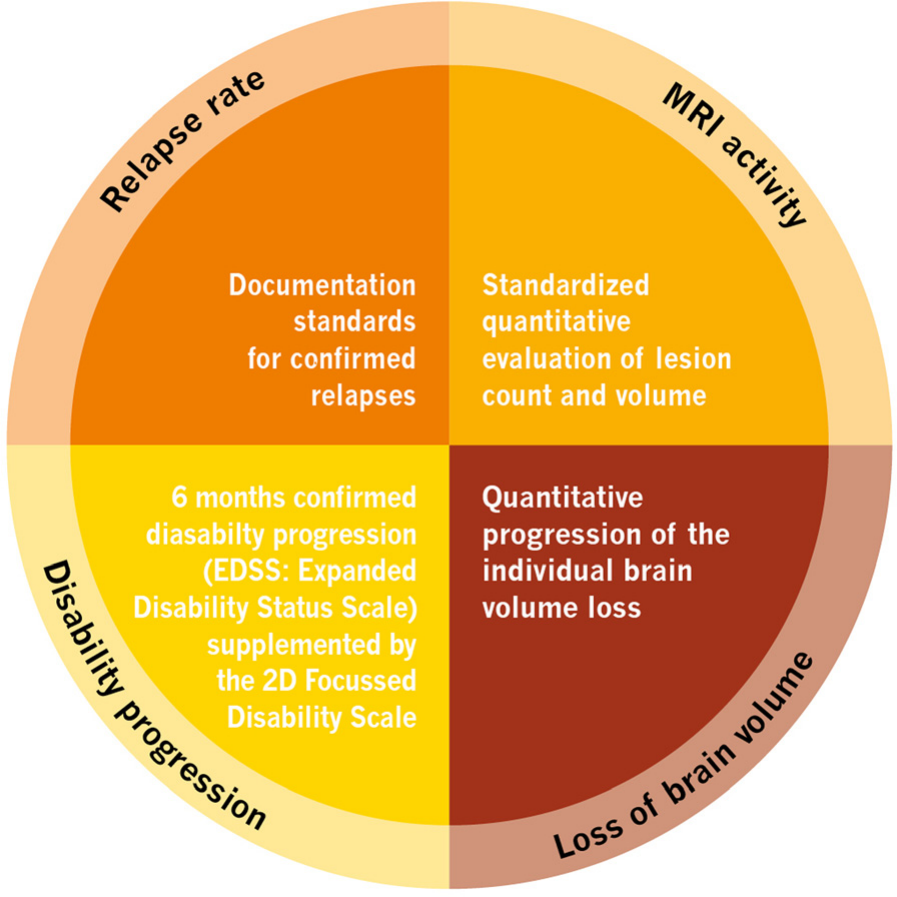
Individual treatment concept NEDA- 4 (no evidence of disease activity-4). This new treatment concept comprises the criteria relapse rate, MRI activity, loss of brain volume, and disability progression (EDSS: Expanded Disability Status Scale). Individual treatment objective, "No evidence of dicease activity-4" (NEDA-4)

**Fig. 4:**
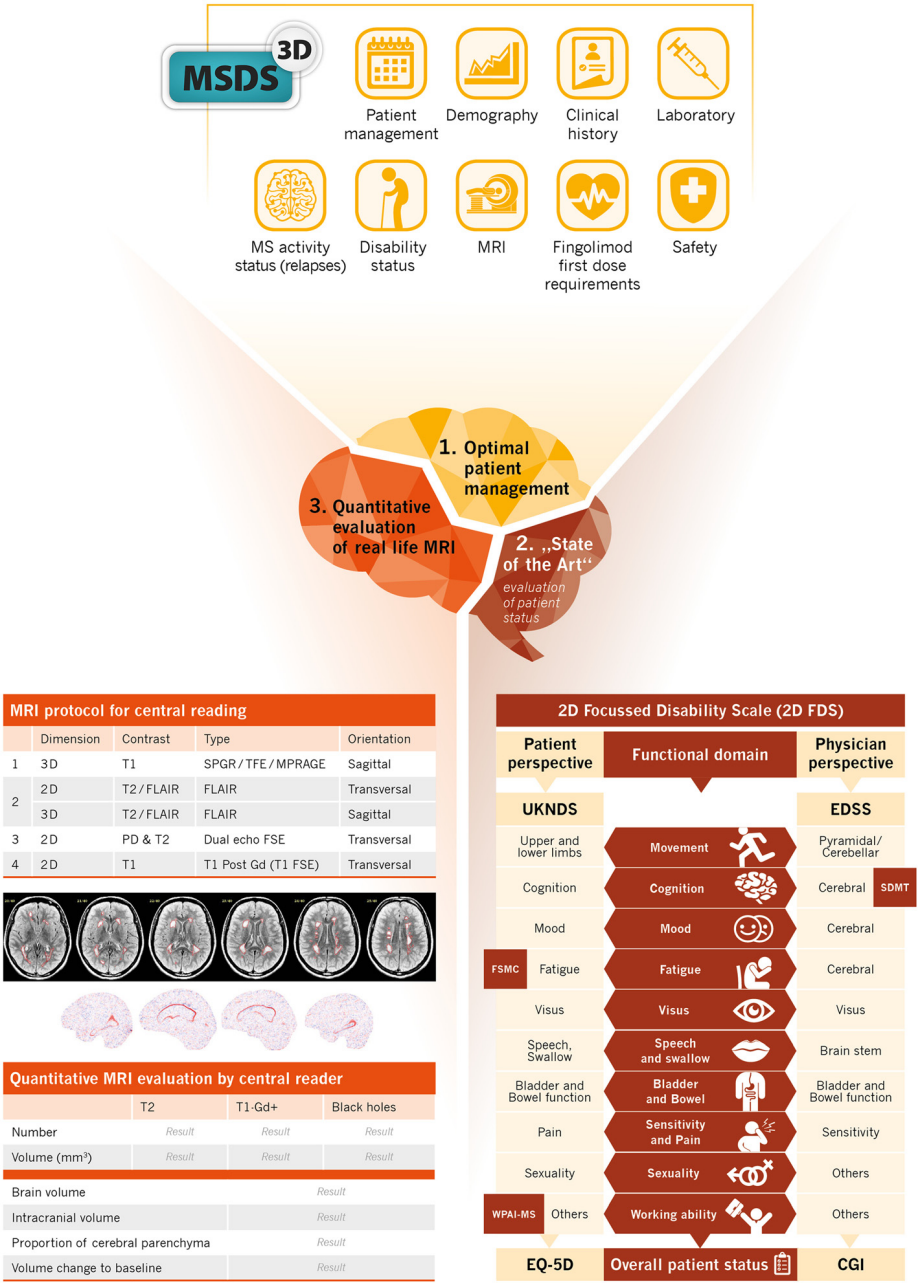
Patient evaluation in PANGAEA 2.0. The software-based MS management system MSDS 3D is supporting patient management (1) and assists physicians in executing all evaluations required for MS diagnosis, treatment initiation, assess safety, and long-term treatment outcome. Patient status (2) is evaluated by means of the 2D Focused Disability Scale (2D FDS) that comprises clinical and subclinical measures representing both the patient’s and the physician’s perspectives (UKNDS: United Kingdom Neurological Disability Scale; FSMC: Fatigue Scale for Motor Fatigue and Cognitive Functions; WPAI-MS: Work Productivity and Activities Impairment; EQ-5D: EuroQuol-5D; EDSS: Expanded Disability Status Scale; SDMT: Symbol Digit Modality Test; CGI: Clinical Global Impression). Standardized quantitative evaluation of routine MRI scans (3) is performed by a central reading facility. The MRI protocol for MRI acquisition and the parameters for quantitative MRI evaluation of lesion load, lesion volume and brain volume are shown. Propose patient evaluation in PANGAEA 2.0

PANGAEA 2.0 is a multicenter non-interventional study (NIS) in RRMS patients who switched to fingolimod (oral, 0.5 mg daily [28]) because of non-responsiveness to or treatment failure with their current therapy. Accordingly, the PANGAEA 2.0 main study is divided in two phases, an evaluation of the current patient status to identify sub-optimally treated RRMS patients and, if these patients are switched to fingolimod, an observational prospective phase of up to 3 years (Fig. 1). The aims of the study are to assess the clinical applicability of the criteria of Lublin [7] to define disease activity as well as the modified Rio score [8] to evaluate treatment response (Fig. 2), and to investigate the long-term benefits of a treatment change to fingolimod, as assessed by parameters that are based on the treatment objectives of NEDA-4 (Fig. 3; [25, 29]). The study further aims at investigating the power of a systematic collection of clinical and subclinical measures that represent the physician’s and the patient’s perspective (2D FDS, Fig. 4). The PANGAEA 2.0 sub-study will additionally evaluate the benefits of standardized MRI analyses obtained from a central reading facility in daily clinical routine. The study started in June 2015 and will end in December 2019. Recruitment will end in December 2016 or after enrollment of 1500 patients who switched to fingolimod (Fig. 1).

PANGAEA 2.0 is conducted in line with the FSA code [30], the joint recommendations of the BfArM (Federal Institute for Drugs and Medical Devices) and the Paul-Ehrlich-Institute on planning, conducting, and evaluating observational studies [31], and the VFA (Research-based Pharmaceutical Companies) recommendations on improving the quality and transparency of NIS [32]. The Ethics Committee of the Dresden University of Technology approved PANGAEA 2.0. The study is registered at the BfArM as NIS 6532 (https://awbdb.bfarm.de).

### Study population

A total of 1500 female and male patients diagnosed with RRMS [33] whose therapy is switched to fingolimod (after evaluation of patient status) are being included in PANGAEA 2.0. In Germany, MS-prevalence is approximately 150 cases per 100,000 residents (122,000 cases; [34]). Approximately 70 % of patients are currently receiving DMTs. In a retrospective analysis [35], 34 % of patients receiving DMTs experienced at least one relapse (28,900). Therefore, approximately 5 % of patients with active disease (1500) are considered to be appropriate to investigate the benefits of a treatment switch to fingolimod. According to data provided by IMS Health, a commercial vendor of prescription drug information (source: IMS Xponent MAT 08/2014), most MS patients (98 %) in Germany are treated in 2800 centers. Therefore, the number of 350 centers participating in PANGAEA 2.0 seems to be sufficient to ensure the representativeness of MS treatment strategies.

For the first phase of PANGAEA 2.0 (evaluation of the patient status), participants are eligible if they were diagnosed with RRMS [33] and have been treated with an approved DMT except fingolimod, or in case of rapidly progressing, severe RRMS, currently untreated patients will also be included. Disease activity is required to be confirmed according to Lublin (Fig. 2; [7]), and patients are required to provide informed consent. To include patients in the second phase of the study, the physician has to decide to switch treatment to fingolimod or to prescribe fingolimod as initial treatment due to high level of disease activity. Prescription of fingolimod or other DMTs is independent of the potential study participation. Reimbursement of physicians was calculated in accordance with governmental regulations and approved by an independent ethics committee. There are no exclusion or selection criteria except for the fingolimod contraindications as listed and described in the product characteristics information [36]. Eligible patients will be enrolled in the sequence in which they present at the physician’s practice.

Only patients who switch to fingolimod will be prospectively followed-up in the 3-year observational phase. Patients not switching to fingolimod after the initial study visit as well as patients who discontinue fingolimod treatment during the 3-year follow-up can be documented as part of the MSDS3D database, but will discontinue study documentation of PANGAEA 2.0.

### Procedures

The study design of PANGAEA 2.0 is outlined in Table 1. In a first phase at visit 0 the current patient status before a potential switch to fingolimod is evaluated. Patients that switch to fingolimod after the initial visit 0 enter a 3 year observational second phase starting with the documentation of the first dose at visit 1. In the observational phase, study visits are scheduled every 3 months (visit 2–14), as recommended by the German Society of Neurology [33].

**Table 1:**
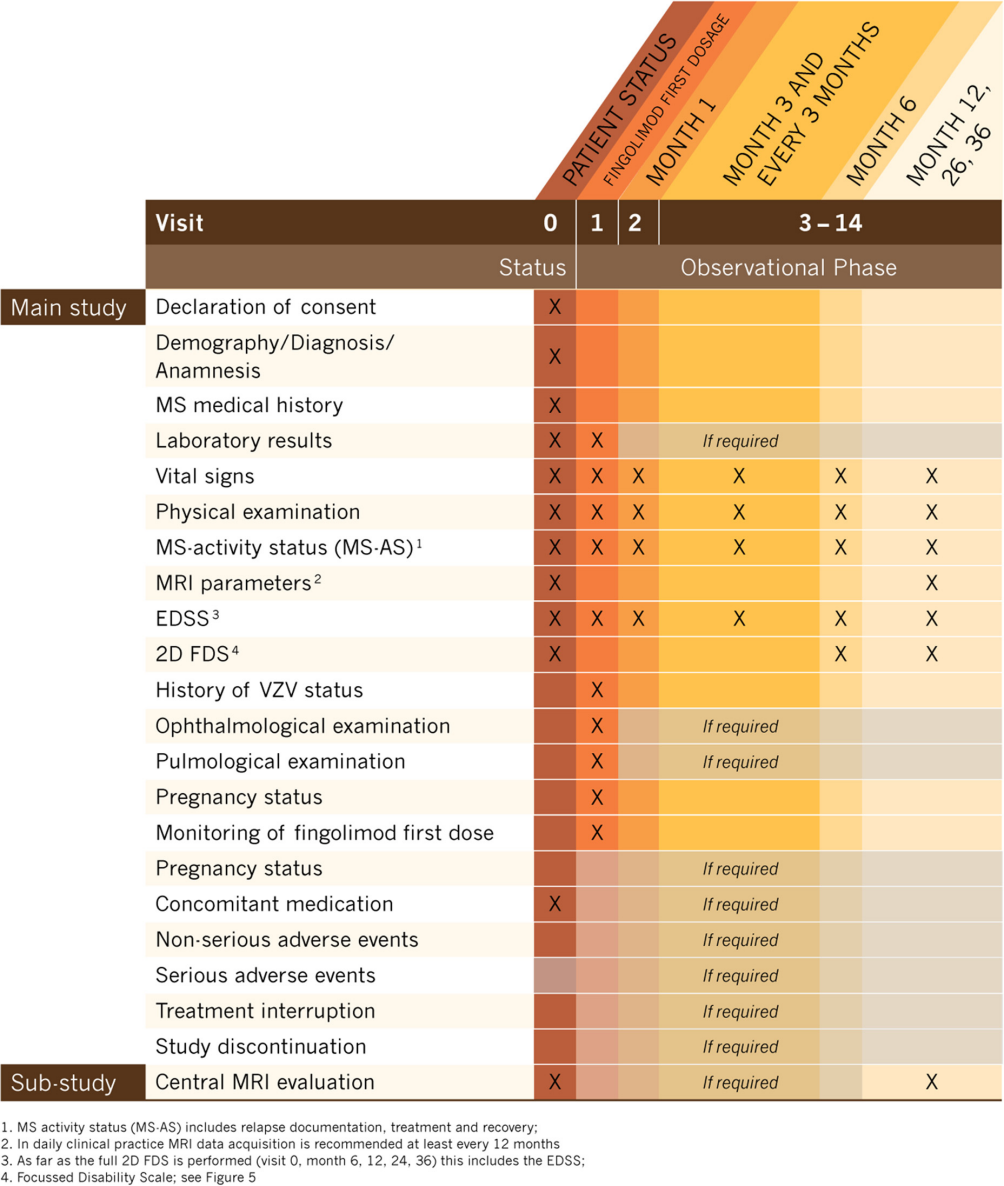
Study visits PANGAEA 2.0

The evaluation of patient status (visit 0) includes the documentation of demographic data, disease history and clinical characteristics, the assessment of disease activity according to Lublin et al. (Fig. 2, Table 1; [7]). It also includes the assessment of a wide range of functional domain parameters (2D FDS; Fig. 4), clinical parameters as well as patient reported outcomes, representing both the physician’s [3, 37, 38] and the patient’s perspectives [39–42].

Patients who are identified as non-responding to or failing treatment with their current therapy and are switched to fingolimod treatment continue documentation in visit 1. According to the summary of product characteristics (SmPC) the switch to fingolimod requires several pre- first dose observations as well as first dose monitoring as described previously [23]. Clinical parameters such as blood cell counts, liver function values, ophthalmological and pulmonological examinations, the documentation and assessment of cardiac diseases and concomitant medication as well as the assessment of the Varicella zoster virus immune status and the pregnancy status are recommended before the first dose of fingolimod and documented in visit1. The monitoring of the first dose of fingolimod includes a 12-channel ECG at before and 6 h after the first dose. Heart rate, blood pressure, and symptoms of bradycardia are examined at 1 h intervals during the post-dose period. In case of the occurrence of clinical relevant cardiac symptoms, clinical management is initiated according to the product information [36, 43]. First-dose monitoring is also required if fingolimod therapy has been interrupted and is re-initiated.

During the 3-year observational phase (visits 2–14 [Fig. 1, Table 1]), data on relapses and disability progression as well as MS activity, including MRI lesions and MS-related BVL are regularly obtained and interpreted according to the modified Rio-score (Fig. 2; [8]) and NEDA-4 (Fig. 3; [25]). For the calculation of the modified Rio-score, a cut-off of four MRI lesions to identify responder/non-responder was applied. In addition, clinical and subclinical measures included in the 2D FDS are evaluated at month 6, 12, 24, and 36 (Fig. 4, Table 1). Premature discontinuation and interruption of therapy along with the reasons therefor and the date of last administration will be documented at any study visit. Investigators will document the occurrence of adverse events at every study visit beginning at visit 1 after switch to fingolimod. Adverse events are defined and will be handled as described previously [23].

In the PANGAEA 2.0 sub-study, 100 MS centers will submit MRI data obtained in accordance with a standardized protocol to a central reading facility (Mediri GmbH, Heidelberg) for qualitative and quantitative evaluation (Fig. 4). Results including data on the number and volume of Gd + T1 lesions, T2/FLAIR hyperintense lesions, new or enlarging hyperintense T2/FLAIR lesions, T1 hypo-intense lesions, and changes of the brain volume will then be reported to the treating physician immediately after evaluation (within approximately 5–7 working days).

### Data management

To collect data and to assist physicians to document and manage all visits and examinations, the MSDS3D will be used [26, 44]. Data will be recorded by the physician or MS nurse responsible either using the web-based MSDS3D electronic case report form or using the locally installed MSDS3D software, both collecting data into the same database. Anonymity of data and content protection are ensured by a complex security process including an encrypted data transfer [45].

Electronic measures of communication facilitate analysis and interpretation of data and are well accepted by patients [45, 46]. The MSDS3D interface displays a vertical timeline and horizontally arranged boxes representing procedures to be executed (e.g., documentation of EDSS, patient questionnaires). The corresponding data input menu can be directly opened from these boxes, and additional procedures can be added to a selected visit. Green color indicates that a procedure has been completed by the MS nurse (e.g., patient questionnaire, SDMT) or the treating neurologist (e.g., EDSS, adverse effects). When all procedures of a visit have been completed, the visit is set as ‘approved’, and data can be transferred to the central PANGAEA 2.0 database. Entries will be automatically controlled for plausibility at the time of data entry and daily reviewed by the database coordinator. All data management processes will be overseen by the data management team of the Clinical Research Organization responsible (Winicker Norimed GmbH Medical Research).

### Statistical analysis

Descriptive statistics will be used for analysis of data. The full analysis set used for analysis includes all patients switching to fingolimod with at least one available post-dose data recording. Median, mean ± standard deviation, minimum, maximum, 5 % percentile, 1st quartile, 3rd quartile, 95 % percentile, number of valid and missing values will be presented in tabular form. For nominal and ordinal-level data, distributions of absolute and relative frequencies will be reported. Incidence rates of all safety outcomes will be evaluated for the patient population switching to fingolimod. For all analyses, the SAS® Version 9.2 will be used.

## Discussion

Here, we report the study design of PANGAEA 2.0, a multicenter NIS on disease active RRMS patients whose therapy is switched to fingolimod. In its first phase, this study evaluates the patient status to support the identification of patients at risk of disease progression. In the second phase, 1500 patients who switch to fingolimod (after the first patient status evaluation) are entering a 3-year observation period. The study is conducted at 350 neurological practices and MS centers in Germany, including 100 centers participating in the PANGAEA 2.0 sub-study on the benefits of a standardized quantitative MRI analysis in daily clinical routine. PANGAEA 2.0 aims not only to expand the fingolimod safety and effectiveness profile, but also to evaluate the applicability of measures for the assessment of treatment response and disease activity in routine clinical conditions.

With the possibility of optimizing individual MS treatment by switching to a more effective medication before severe neurological deterioration occurs the identification of non-responders to the current MS therapy has gained fundamental importance. However, without a standardized definition of non-responders for clinical routine, the decision when to switch therapy is challenging. Prediction of treatment efficacy based exclusively on the traditional measures relapse rate and EDSS progression has been shown to be of limited value [47]. Since frequent MRI has been demonstrated to predict non-response to IFN-β at early stages [48–52], Rio et al. proposed a combined assessment of clinical relapses, EDSS progression, and active MRI lesions after 1 year of treatment [52]. Based on the observation that patients who were positive for two of these three parameters had a higher probability of disability progression and relapse activity, Sormani et al. [8] proposed the modified Rio Score (Fig. 2). The modified Rio score combines short-term changes (during 1 year of IFN-β treatment) in relapse frequency and MRI lesions as a surrogate marker for long-term disability progression [8, 53]. Patients are classified as high, medium, and low-risk patients according to the number of relapses and new T2 lesions within 1 year of IFN-β treatment. Medium-risk patients are then re-assessed after 1.5 years of treatment [54]. The modified Rio score then allows to identify patients at risk for non-responding to IFN-β treatment in the long-term [10].

In 2014, Lublin et al. [7] refined the established MS phenotypes by adding disease activity as an additional descriptor of MS pathogenesis. Active disease is defined by relapses, acute or sub-acute episodes of new or increasing neurological dysfunction during the previous 12 months, or contrast enhancing T1 or new or unequivocally enlarging T2 hyperintense lesions. In PANGAEA 2.0, the criteria of Lublin will be employed to assess disease activity at visit 0 in order to identify patients at risk of non-response, while the modified Rio score will be used to evaluate treatment response during the first year of treatment (Fig. 2). Signs of disease activity or progression might then indicate the need to initiate therapy of treatment-naïve patients or to switch therapy of patients who are not responding to their current medication. Since the modified Rio score has not been used to identify patients who are not responding to therapies other than IFN-β, PANGAEA 2.0 will provide further insights into the applicability of this score to evaluate treatment response to fingolimod in clinical routine.

NEDA has evolved both as a concept for treatment success of individual MStreatment [55] and as an outcome measure of DMTs in clinical trials [56]. Cohen et al. [57] assessed the proportion of IFN-β1a and fingolimod-treated patients who achieved NEDA after 1 year and 2 years of treatment (defined as no relapses, no 3-month confirmed disability progression, and no MRI activity) and found a higher NEDA-proportion among fingolimod-treated patients than among IFN-β1a-treated patients, as well as an increased NEDA-proportion among the IFN-β1a group after the switch to fingolimod. Importantly, the authors demonstrated the value of NEDA assessment during the first year of treatment for the prediction of long-term outcomes. However, there is still controversy with regards to the most relevant measurements for the assessment of treatment response in RRMS patients. Other scores using different algorithms taking disability progression, relapses, and MRI assessments into account to evaluate a treatment response have also been proposed [11, 12, 25, 58].

Due to the complexity and the heterogeneous course of the disease, additional outcome measures such as BVL have been suggested to complement the NEDA criteria. BVL begins at early MS-stages and is associated with disability progression and cognitive decline [59–62]. Since treatment effects on BVL correlate with those on disability progression, this parameter might provide predictions of future outcomes [56, 63]. In this study, we will therefore assess treatment response according to the NEDA-4 parameters (relapse rate, MRI activity, BVL, disability progression [Fig. 3]) as well as subclinical measures summarized in the 2D FDS (Fig. 4). Since deterioration not only occurs in the motor, visual, and sensory systems, this scale additionally includes cognitive changes, mood swings, fatigue, bowel and bladder function, sexual dysfunction, quality of life, as well as work productivity and activity to obtain a comprehensive picture of the patient’s disease status [64]. All the above measures,, are integrated into our proposed approach for patient evaluation aimed to comprise optimal patient management, ‘state of the art’ evaluation of patient status, and quantitative evaluation of MRI in daily clinical practice (Fig. 4).

For the assessment of NEDA-4 parameters a frequent MRI monitoring (e.g., annual), carried out under standardized conditions is required. We have therefore planned a sub-study that evaluates the value of routine MRI scans performed in accordance with a standardized protocol to ensure comparability of results. MRI scans are examined by a central reading facility to consistently obtain quality controlled standardized quantitative results according to lesion number, lesion volume and brain volume (Fig. 4).

The processing of large quantities of data obtained in this study demands intense data management [44]. The MSDS3D software already employed in the predecessor study PANGAEA [23] allows the documentation and management of visits and examinations as well as the integration of data input from different sources. The MSDS3D-PANGAEA 2.0 module will assist physicians in all processes required for the identification of patients at risk of disease progression and potential fingolimod switch patients (Fig. 4; [26, 43]).

The main objective of PANGAEA 2.0 is to expand the knowledge on the safety and effectiveness of a switch to fingolimod in RRMS patients who are not-responding to or having treatment failure with their current MS medication. In the predecessor study, PANGAEA, most RRMS patients who started fingolimod (Gilenya®) therapy had been pretreated with injectable BRACE therapies (Betaferon®, Rebif®, Avonex®, Copaxone®, Extavia®) or natalizumab (Tysabri®). Few patients were treatment naïve at the time of inclusion or pretreated with Azathioprine (Imuran®)/Mitoxantrone (Novantron® and Mitoxantron Ebewe®). Other substances such as alemtuzumab (Lemtrada®), dimethyl fumarate (Tecfidera®), and teriflunomide (Aubagio®) have been approved since the end of the PANGAEA recruitment phase. PANGAEA 2.0 will therefore provide new information on the safety of fingolimod in RRMS patients pretreated with these therapies in routine clinical practice. Furthermore, the predecessor study, PANGAEA [23], focused on the post approval fingolimod safety profile, comprising precautions to treatment and first dose monitoring, as well as on parameters regarding global symptomatology (CGI [38]) and disability (EDSS [3]). PANGAEA 2.0 will hence add useful information on the effectiveness of fingolimod by additionally assessing early and subtle signs of disease activity, including data on MRI activity and BVL, as well as on subclinical changes in, for example, cognition, fatigue, and activity (Fig. 4). Valuable data on comparative DMT effectiveness have recently been obtained by registry-based research such as MSBase analyses [65–68]. Since registry- and trial-based research are subject to different requirements, the comparison of results will provide additional information on the effectiveness of different treatment algorithms.

In summary, PANGAEA 2.0 will assess not only the long-term benefit of a treatment change to fingolimod, but also the applicability of clinical and subclinical parameters and definitions for the assessment of disease activity, as defined by Lublin et al., disability progression and treatment response evaluated by the he modified Rio score, the definition of individual treatment concepts according to NEDA-4, and the clinical and subclinical measures of 2D FDS [69]. The data to be obtained in PANGAEA 2.0 will expand the existing safety and effectiveness profile of fingolimod and will contribute to the establishment of novel concepts of decision making in MS treatment.

## Abbreviations

2D FDS, 2D Focussed Disability Scale; BfArM, Federal Institute for Drugs and Medical Devices; BVL, brain volume loss; CGI, Clinical Global Impression; DMT, disease-modifying therapy; EDSS, Expanded Disability Status Scale; EQ-5D, EuroQuol-5D; FSMC, Fatigue Scale for Motor Fatigue and Cognitive Functions; IFN, interferon; MRI, magnetic resonance imaging; MS, Multiple Sclerosis; NEDA-4, no evidence of disease activity-4; NIS, non-interventional study; PANGAEA 2.0, Post-Authorization Non-interventional GermAn treatment benefit study of GilEnyA in MS patients; RRMS, relapsing-remitting MS; SDMT, symbol digit modality test; SmPC, summary of product characteristics; UKNDS, United Kingdom Neurological Disability Scale; VFA, Research-based Pharmaceutical Companies; WPAI-MS, work productivity and activities impairment
